# Evaluation of Antioxidant Potential of Commercial Cinnamon Samples and Its Vasculature Effects

**DOI:** 10.1155/2022/1992039

**Published:** 2022-03-23

**Authors:** Emily K. G. Moreno, Isaac Y. L. de Macêdo, Erica A. Batista, Fabio B. Machado, Gabrielle R. Santos, Daniela M. L. Andrade, Matheus L. Rocha, Nerilson M. Lima, Boniek G. Vaz, Eric S. Gil

**Affiliations:** ^1^College of Pharmacy, Federal University of Goiás (UFG), 74605-170, Brazil; ^2^Institute of Chemistry, Federal University of Goiás (UFG), 74690-900, Brazil

## Abstract

Growing concerns on free radicals are the oxidative processes associated with physiological damage. The consumption of functional foods and use of plants with antioxidant capacity are widespread. Given the importance of determining antioxidant capacity in relation to the therapeutic effect, this study was aimed at evaluating cinnamon extract (*Cinnamomum* sp.) in commercial samples by spectrophotometric and voltammetric methods and assessing the vascular activity of some samples. The spectrophotometric methods performed were DPPH (1,1-diphenyl-2-picrihydrazine), ABTS (2,21-azinobis-(3-ethylbenzothiazoline-6-sulfonic acid)), and Folin-Ciocalteu radical sequestration assays. For the electrochemical experiments, a three-electrode system was used, consisting of carbon paste electrode, platinum wire, and Ag/AgCl/KCl_sat_, representing the working, auxiliary, and reference electrodes, respectively. The electroanalytical methods used were differential pulse, square wave, and cyclic voltammetries. The extracts were prepared in hydroalcoholic solution. A calibration curve with gallic acid was calculated to quantify their equivalent amounts in the analyzed extract. The correlation between the electrochemical approach and the total phenols calculated by the ABTS, DPPH, and Folin-Ciocalteu methods was 0.63, 0.7, and 0.73, respectively, with 1 being an ideal directly proportional correlation. The correlation between spectrophotometric methods was 0.83. A biosensor was developed in a carbon paste electrode using the enzyme laccase, obtained by the fungus *Marasmiellus colocasiae*. It was observed that the antioxidant profile of the cinnamon samples had an analytical sign improvement of up to 4 times when compared with the electrode without the modification. The samples were analyzed by mass spectrometer, and the main chemical markers found were coumarin, cinnamaldehyde, and eugenol. Pharmacological trials showed that these samples also induce a significant vasorelaxant effect associated to antioxidant potential on vascular injury induced by oxidative stress. Thus, cinnamon showed a high antioxidant capacity, in agreement with the results obtained in other studies, emphasizing its importance as a functional food.

## 1. Introduction

Reactive oxygen species (ROS) are highly reactive compounds formed from successive reductions of O_2_ present in the body's natural physiological functions such as regulation of cell growth, energy production, and phagocytosis [1]. This group includes the hydroxyl radical (^•^OH), superoxide anion (O_2_^•-^), and hydrogen peroxide (H_2_O_2_) that in excess in the body are related to several health prejudice, such as degenerative diseases, lipid peroxidation and damage in enzymes, proteins, carbohydrates, and DNA [2–4]. Moreover, up to 70% of the ROS produced can be transformed to reactive chlorine species such as hypochlorous acid (HOCl) and hypochlorite (OCl^−^) in the vasculature through the action of some enzymes (vascular peroxidase 1 and myeloperoxidase), leading to greater injuries in the cardiovascular system [5, 6].

A strategy for decreasing ROS in the body is the intake of antioxidants that are compounds capable of preventing oxidative degradation reactions through the stabilization of radical compounds [7]. The addition of antioxidants from natural origin in food products has become increasingly popular due to its importance in improving nutritional conservation and the quality of life for consumers [8, 9].

Cinnamon species belongs to the *Lauracea* family group that has eugenol and coumarins in their composition, which are phenolic compounds with high antioxidant power. Moreover, it also has proven antimicrobial activity due to the components cinnamaldehyde, cinnamic acid, aromatic aldehyde, benzoic acid, and benzaldehyde present in its structure [10–13].

The antioxidant capacity of natural products, whether of extract or isolated compound, is traditionally analyzed by spectrophotometric methods such as the radical 1,1-diphenyl-1-picryl-hydrazil (DPPH), the radical 2,2′-azinobis-(3-ethylbenzothiazoline-6-sulfonic acid) (ABTS), and Folin-Ciocalteu, which relate the discoloration of radicals with the antioxidant power of the analyzed product [14–17].

Although colorimetric methods are well established in the literature, they have several limitations, mainly related to interferences of molecules that absorb in the same range. In addition, there is a need for efficient prepreparation of the samples, since factors such as precipitation, suspension of large particles, and the opacity of the medium alter the absorbance reading [18, 19].

Comparatively, electroanalytical methods are a promising alternative for analyzing the antioxidant capacity of complex samples due to their high sensitivity, analysis speed, low cost, low reagent consumption, and generally nontoxicity. This technique is based on the correlation between the redox reaction of the antioxidant capacity and its detectable electrical properties [20, 21].

An alternative to improve the electrochemical results is the development of a biosensor, which uses biological reactions to detect specific components of a sample [22, 23]. A biosensor can be formed by an enzyme such as laccase that coupled to a transducer results in a signal proportional to the concentration of the analyte investigated [24–26].

Given the importance of determining antioxidant capacity in relation to the therapeutic effect, this study was aimed at evaluating cinnamon extract (*Cinnamomum* sp.) in commercial samples by spectrophotometric and voltammetric methods.

## 2. Materials and Methods

### 2.1. Reagents and Natural Products

All electrolyte solutions were used with analytical purity and were diluted in distilled Milli-Q water (conductivity ≤ 0.1 *μ*S.cm^−1^) (Millipore S. A., Molsheim, France). Gallic acid, ethanol, and the standards of DPPH, ABTS, and Folin-Ciocalteu were purchased from Sigma-Aldrich Chemical Co. (St. Louis, MO, USA).

Seventeen commercial samples of powdered cinnamon were acquired from local businesses in Brazil and other countries such as Costa Rica, Greece, Portugal, Spain, and the United States of America.

### 2.2. Sample Preparation

Extraction fractions were prepared with 100 mg of the commercial samples of powdered cinnamon and 10 mL of different proportions of water and ethanol, being 100 : 0, 70 : 30, 50 : 50, 30 : 70, and 0 : 100 (water/ethanol), respectively. The samples were vortexed for 1 min, followed by 10 min in ultrasound and 5 min in centrifugation at 2500 rpm.

In order to avoid the presence of ethanol in the pharmacological assays (which could damage the vascular tissues), the samples 7 and 15 were lyophilized and resuspended in distilled water (100 mg/mL). Lyophilization was performed in a 25 L Genesis SQ Super XL-70 lyophilizer from SP Científica. For the procedure, the sample was frozen at -60 degrees at a pressure of 380 Torr (0.5 bar). After the sample was completely frozen, the pressure was reduced to 100 mTorr (0.0001333 bar) and held at that pressure and temperature for 72 h. Then, the temperature was increased to -10 degrees, keeping the same pressure and temperature for 72 h.

### 2.3. Electroanalytical Tests

The voltammetric experiments were performed in *μ*Autolab III® potentiostat/galvanostat integrated with NOVA 2.1 software (Metrohm). The measurements were made in a 5 mL single compartment electrochemical cell with a 3-electrode system, consisting of a carbon paste electrode, an Ag/AgCl/KCl_sat_ 3 M electrode, and a platinum wire (purchased from Lab Solutions, São Paulo, Brazil), representing the working, reference, and auxiliary electrode, respectively. The experimental conditions for DPV were pulse amplitude of 50 mV, pulse width of 0.5 s, and scan rate of 10 mV.s^−1^. The experimental conditions for the SWV were pulse amplitude of 50 mV with a frequency of 50 Hz and a potential increase of 2 mV, corresponding to a scanning rate of 100 mV.s^−1^. The experimental conditions for CV were scan range from 0 to 1 and scan rate of 100 mV.s^−1^. The DPV voltammograms were corrected with the baseline-corrected, and all data were analyzed and treated in the Origin 9.0 software.

All experiments were done at room temperature (21 ± 1°C) in triplicate (*n* = 3), and the main electrolyte used was 0.1 M phosphate buffer (PB) pH 7.0.

### 2.4. Electrochemical Index

The electrochemical index (EI) takes into account major voltammetric parameters, such as anodic peak potential (Epa) and anodic peak current (Ipa). Based on the fact that in lower Epa, the electron donation ability is greater (thermodynamic parameter) and the higher the Ipa (kinetic parameter), the greater the number of electroactive species, EI is calculated using the following:

(1) $$\begin{array}{c} \text{EI}=\overset{n}{\underset{i=1}{\sum }}\frac{\text{Ipan}}{\text{Epan}} \end{array}$$

where in a voltammogram with *n* peaks, Ipan and Epan are the current and the potential of the *n* peak, respectively.

### 2.5. Biosensor

The fungus *Marasmiellus colocasiae* CCIBT 3388, isolated in Domingos Martins-ES/2005, was obtained from the Basidiomycete Culture Collection (CCB) of the São Paulo Institute of Botany. The identification of the obtaining source ex situ of genetic heritage, with the information contained in the records, is in accordance with § 1 of the Article 22 of the Decree No. 8.772 of 2016. The crude enzymatic extract from *Marasmiellus colocasiae*, developed as described in previous work of our research group [27], was used to prepare the optimized biosensor.

Briefly, 50 *μ*L of enzymatic crude extract was mixed with 70 mg of graphite (Sigma-Aldrich®) and left to dry at room temperature for 1 h. Then, 30 mg of mineral oil, the agglutinating agent, was added and rigorously mixed in order to achieve a homogenous paste. This final paste was used to fill the cavity of the electrode that was 2 mm in diameter and 0.5 mm in depth. The experimental conditions for DPV were pulse amplitude of 50 mV, pulse width 0.5 s, scan rate of 10 mV.s^−1^, and scan range from 1 to 0 V. All experiments were done at room temperature (21 ± 1°C) in triplicate, and the main electrolyte used was 0.1 M sodium acetate buffer, pH 5.0.

### 2.6. DPPH Radical Scavenging Assay

The radical scavenging activity was done using the DPPH reagent, according to the well-established procedures [24]. A mixture of 2.7 mL of DPPH ethanolic solution (0.1 mM) and 0.3 mL of ethanol was prepared as a blank control, where the final absorbance at 517 nm was a.c. 0.7. Ethanol was used to adjust the baseline (*A* = 0.00).

For the experiments, 300 *μ*L of ethanolic portion was replaced by the extract and by the standards when the calibration curve was performed. The measurements were analyzed using UV-vis spectrometer (model V-530, Jasco, Inc., Easton, MD, USA). Each trial was performed in triplicate. The antioxidant activity was expressed as IC50 as indicated in Equation (2), where 50% of the sample solution is capable of producing discoloration compared to the white control (ethanol) after five min of transferring the sample aliquot to the DPPH radical. The samples were analyzed in a 1 cm optical path cuvette at room temperature.

(2) $$\begin{array}{c} \%\text{AA}=\frac{\text{ADPPH}-\text{Atest}}{\text{ADPPH}}\times 100. \end{array}$$

where % AA is the percentage of antioxidant activity and ADPPH is the absorbance of the solution with the radical formed without the presence of a sample. Atest is the absorbance observed in the presence of the radical with the analyte, and IC50 is the amount of extract in g/mL of the tested samples needed to decrease the initial ABTS concentration by 50%.

Linearity was observed in the calibration curves of gallic acid with the regression equation.

### 2.7. ABTS Radical Scavenging Assay

The ABTS radical assay was conducted according to literature [20], formed from the reaction of 5.0 mL of ABTS solution in water (7 mM) with 88 *μ*L of potassium persulfate solution in water (140 mM), and incubated in the absence of light for 16 hours. Then, 2 mL of the prepared radical solution was diluted in ethanol to 150 mL, obtaining a solution with an absorbance a.c. 0.7 at a wavelength of 734 nm.

For the experiments, 300 *μ*L of the ethanol extract was added to the test tube containing 2.7 mL of ABTS radical; thus, the tubes were covered with Parafilm® and kept in the dark for 20 min. The absorbance was monitored with the same spectrophotometer used for DPPH assay. All tests were performed in triplicate. The decay percentage expressed by absorbance at 734 nm was calculated as

(3) $$\begin{array}{c} \%\text{AA}=\frac{\text{AABTS}-\text{Atest}}{\text{AABTS}}\times 100, \end{array}$$

where % AA is the percentage of antioxidant activity and AABTS is the absorbance of the solution with the radical formed without the presence of a sample. Atest is the absorbance observed in the presence of the radical with the analyte, and IC50 is the amount of extract in g/mL of the tested samples needed to decrease the initial ABTS concentration by 50%.

Linearity was observed in the calibration curves of gallic acid with the regression equation.

### 2.8. Total Phenolic Assay

The total phenolic compounds were determined by Folin-Ciocalteu (FC) spectrophotometric method. Cinnamon samples at a concentration of 1% were prepared, and aliquot of 50 *μ*L of the extract was added in a test tube containing 1 mL of distilled water and 250 *μ*L of the FC reagent. After 5 min, 750 *μ*L of a 20% Na_2_CO_3_ solution and 2950 *μ*L of distilled water were added. The mixture was incubated in the absence of light for 60 min, obtaining absorbance of a.c. 0.7 at a wavelength of 765 nm. The quantification of phenolic compounds in cinnamon samples was carried out in triplicate and expressed by means of gallic acid equivalents in *μ*M, from a calibration curve obtained under the same conditions for sample analysis [28, 29].

### 2.9. Mass Spectrometry Analysis

A metabolic assessment of cinnamon samples was performed by direct injection of untreated samples into a mass spectrometry system. The samples were dissolved in methanol at concentration of 500 ppm, filled into a 500 *μ*L syringe (Hamilton) and infused directly using a syringe pump at a flow rate of 3 *μ*L/min, and analyzed on an Q-Exactive Orbitrap (Thermo Scientific, Bremen, Germany) equipped with an electrospray ionization (ESI) source. The analyses were performed in negative (ESI (-)-MS) and positive (ESI (+)-MS) mode and mass range of 100-1000 Da. The identification of the main metabolites detected in the cinnamon samples was performed using online dereplication platforms (MetFrag, PubChem, and CFM-ID).

### 2.10. Studies in Isolated Arteries

Male Wistar rats (7-8 weeks old, 210–240 g) from the Central Bioterium of the Federal University of Goiás were used in this protocol. All experiments were carried out in agreement with the Brazilian Society of Laboratory Animal Science and were approved by the Local Ethics in Research Committee (Protocol CEUA/UFG 116/19). The rats (*n* = 5 − 7 for each different protocol) were anaesthetized and killed (cardiac puncture and exsanguinations), and the thoracic aorta was removed, cleaned and cut into rings (±3-4 mm in length), placed in an organ bath (10 mL) between two stainless-steel stirrups, and connected to a computerized system and a WinDaq Resource (DATAQ Instruments, Akron, OH, USA) data acquisition unit to measure vascular isometric tension. The isolated vascular chamber was completed with physiological salt solution with the following composition: 130 mM NaCl, 1.2 mM KH_2_PO_4_, 4.7 mM KCl, 14.9 mM NaHCO_3_, 1.2 mM MgSO_4_, 1.6 mM CaCl_2_ and 5.5 mM glucose, at pH 7.4, and gassed with carbogenic gas (95% O_2_ and 5% CO_2_) at 37°C.

The vascular rings were firstly stretched to a basal tone of 1.5 g before allowing them to equilibrate in the bathing solution. The endothelial cell integrity was demonstrated by the presence of relaxation (minimum 80% of relaxation) to acetylcholine (1 *μ*M) after being precontracted with phenylephrine (0.1 *μ*M). After equilibration, cumulative concentration-response curves for the cinnamon samples (0–380 *μ*g/mL, lyophilized and diluted in distilled water) were generated using isolated aortic rings with functional endothelium that had been precontracted with phenylephrine (0.1 *μ*M). For the vascular studies, only the cinnamon samples that showed the best and the worst antioxidant activity were used (samples 7 and 15, respectively). Furthermore, in order to verify the relative contribution of endothelium nitric oxide to the vascular relaxation induced by cinnamon samples, the same protocols were repeated after treatment (30 min) with nitric oxide synthase inhibitor (L-NAME, 100 *μ*M).

In other series of experiments, the vascular contraction induced by adrenergic agonist (phenylephrine, 0.1 *μ*M) was investigated in control vessels or after oxidative stress stimulus characterized by the addition of hypochlorite (OCl^−^) in the bath solution at concentration of 5 *μ*M (60 min) [6, 30]. After 60 min, the arteries with intact endothelium were contracted again with phenylephrine (0.1 *μ*M), and the level of contraction was measured. In addition, some arteries were treated with cinnamon samples (samples 7 and 15) in two different concentrations (100 and 500 *μ*g/mL) at the same time as the oxidative stress stimulus with OCl^−^ (treatment with 5 *μ*M OCl^−^ plus cinnamon samples for 60 min).

### 2.11. Statistical Analysis

For the electrochemical and radical scavenging assay data analysis, the Origin 9.0 statistical software was used. A correlation matrix was performed for the antioxidant parameters evaluated, i.e., ABTS, DPPH, Folin-Ciocalteu, and EI. ANOVA analysis was performed for the unmodified and modified electrode assays with the null hypothesis of equal averages and 0.05 of significant value.

In the vascular studies, values were expressed as mean ± SEM. Comparisons among groups were performed using one-way ANOVA (posttest: Newman–Keuls) with 0.05 of significant value. Analyses were carried out using GraphPad Prism version 5.00 for Windows (San Diego, CA, USA).

## 3. Results and Discussion

### 3.1. Solvent Extraction

Cinnamon is constituted by several components of different polarities [31]; for this reason, water and ethanol were evaluated to investigate the effectiveness of cinnamon extraction using a pool of all samples. Extractions were analyzed through the EI (*μ*A/V) of the cinnamon for each solvent (Table 1).

Samples with higher concentration of ethanol demonstrated better extraction than aqueous solution because ethanol can be considered a bipolar solvent that dissolves most slightly nonpolar and polar organic compounds that make up most of the molecules with antioxidant action [32].

The result obtained in this work corroborates with the literature that describes a better cinnamon extraction with a combination of both solvents than when used separately [33]. The solvents of water/ethanol in the proportions of 70 : 30 (*v*/*v*), 30 : 70 (*v*/*v*), and 0 : 100 (*v*/*v*) presented EI higher than 50 *μ*A/V without significant statistical differences. Therefore, the solvent water/ethanol 30 : 70 (v/v) was chosen for the further experiments.

#### 3.1.1. *Cinnamon* Characterization

**Figure 1:**
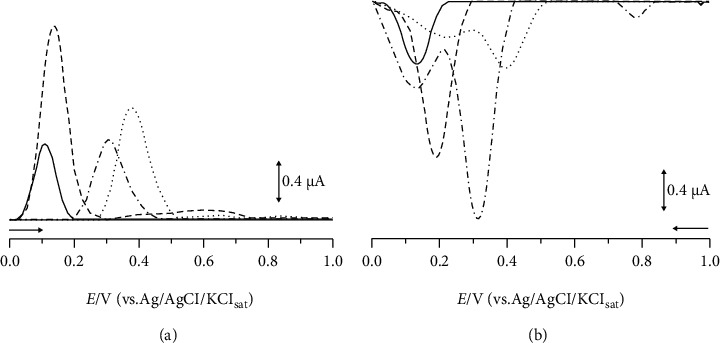
DP voltammograms of different pHs obtained for nonmodified carbon paste electrode in 0.1 M phosphate buffer (a) and biosensor with 0.1 M sodium acetate buffer (b) for cinnamon. For pH 9.0 (––––), pH 7.0 (– – –), pH 5.0 (– - –), and pH 3.0 (......).

In order to evaluate the redox profile of *Cinnamon* samples, electrochemical experiments were performed with a pool of all samples. The electrolytic support was studied from pH 3.0 to pH 9.0 (Figure 1). This is an important parameter to be evaluated, once it allows the electron flow for the passage of current in the reaction medium [34].

The nonmodified carbon paste electrode presented a better resolution and sensitivity in the pH 7.0, in agreement with Souza-Sartori [35], which demonstrated that phenolic compounds are better solubilized in a pH range of extraction between 6.0 and 8.0. For the modified electrode with the laccase enzyme obtained from the fungus *Marasmiellus colocasiae*, the signal increased in the pH 5.0. According to literature, laccase enzyme is often used in pH conditions ranging from 3.5 to 5.5 [36, 37]. Therefore, these electrolytic supports were used in the further analysis.

**Figure 2:**
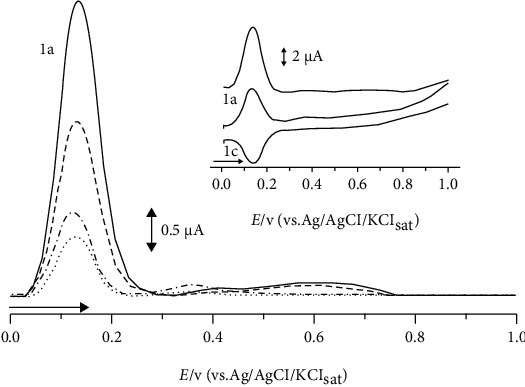
Differential pulse voltammetry (DPV). Inset: square wave voltammetry (SWV) of cinnamon commercial samples (0.01%). Samples A (––––), B (– – –), and C (– - –) and second scans (.......).

Figure 2 shows the voltammetry profiles of commercial cinnamon samples.

Among the evaluated samples, a similar profile was observed, where an anodic peak c.a.E_pa1_ = 0.1 V was present. Anodic peaks in potentials c.a.E_pa1_ = 0.2 V or in lower values are indicative of polyphenol compounds [38]. Due to the low overpotential values seen in this compounds' oxidation process, it can be inferred that these compounds have high antioxidant capacity and the same can be said about the other samples [24].

The intensity of the peak varied in a decreasing order from samples A, B, and C. These samples were chosen because they represent the highest, median, and lowest results of EI, originated from Paraná (BRA), Goiás (BRA), and Lisboa (PRT), respectively. Since the intensity of the anodic peak is correlated with the concentration of the sample by the Cottrell equation [39], sample A likely has the highest amount of antioxidant compounds and sample C likely has the lowest amount.

The reversibility of the anodic peak c.a.E_pa1_ = 0.1 V was also observed for all sample analysis, corroborating the presence of polyphenol compounds in these samples. Moreover, samples A and B presented a second slight anodic peak c.a.E_pa2_ = 0.6 V and sample C in c.a.E_pa2_ = 0.4 V; these anodic peaks can be correlated to other phenolic compounds present in lesser quantities [20, 40].

### 3.2. Antioxidant Capacity

The variables of antioxidant capacity were compared and evaluated by the spectrophotometric methods ABTS, DPPH, and Folin-Ciocalteu with electrochemical experiments through the EI values for all samples. A correlation matrix was calculated (Table 2).

A high correlation coefficient of approximately 0.7 was seen between EI and DPPH/ABTS/Folin-Ciocalteu, which shows that despite the difference in methodologies, being the first electroanalytical and the latter scavenging, the inferences about the redox process of the antioxidant capacity are closely related. The EI correlation between modified and nonmodified electrodes was 0.62. Moreover, the correlation coefficient between ABTS and DPPH was also high (c.a. 0.8).

The total phenol of cinnamon was expressed by gallic acid equivalents. Hence, a calibration curve with concentrations from 4.97 *μ*M to 166.66 *μ*M for gallic acid was constructed in order to get the linear equation *Y* = 4.34e^−7^ + 1.20e^−8^*X* (intercept SD = 6.10^e−23^, slope SD = 5.68e^−25^), obtaining *R*^2^ = 0.99. The samples of cinnamon with greatest gallic acid quantity were samples 3 and 7, corresponding to 0.17 and 0.19AG (g/g ext), respectively (Table 3).

According to the Folin-Ciocalteu method, total phenols are more present in the cinnamon samples 7 and 8, which corroborates with the electrochemical studies that also showed a higher EI for the sample 7. Likewise, the results obtained for both methodologies also showed agreement for the sample with the lowest antioxidant capacity, represented by the cinnamon sample 14.

### 3.3. Biosensor

Table 4 shows the results obtained from the carbon paste electrode modification using the laccase enzyme of the fungus *Marasmiellus colocasiae*.

The enzymatic biosensor of *Marasmiellus colocasiae* produces biochemical oxidation followed by an electrochemical reduction detected amperometrically that allows the detection of polyphenols. Thus, the modified electrode showed improvement in all samples, with a signal increase of up to 4 times, due to the laccase's ability to oxidize the phenolic compounds present in the cinnamon [41, 42].

Corroborating the spectrophotometric data of the Folin-Ciocalteu assay with the *Marasmiellus colocasiae* biosensor, the biosensor presented better results in 7 and 8, confirming the previous results in the other electrochemical and spectrophotometric methods, through the amplification of the signal promoted by the enzyme, used as an auxiliary electrochemical tool in the detection of phenolic compounds present in cinnamon [27].

### 3.4. Mass Spectrometry Analyses

The purpose of this study was to annotate compounds that contribute to antioxidant potential from cinnamon samples based on their mass to charge ratios (*m/z*) and elemental composition obtained through Orbitrap mass analyzer, as well as chemotaxonomy data reported in literature. Hence, cinnamon samples were analyzed by high-resolution mass spectrometry (HRMS) in order to identify the main chemical markers and associate with their antioxidant activity. A wide range of phenolic compounds such as flavonoids, coumarin, phenolic acids, and cinnamic acid derivatives were annotated by MS analyses, which have recognized antioxidant activity [43]. Fifteen antioxidant compounds reported in cinnamon were annotated in the current study, conforming Table 5.

The major peak in terms of % area peak from the cinnamon samples belonged to coumarin at *m/z* 147.04. The applied spectrometric study allowed inferring the substances responsible for antioxidant potential from cinnamon samples. Therefore, mass spectrometry is a rapid and sensitive technique that can be applied to the identification of bioactive compounds in cinnamon samples.

**Figure 3:**
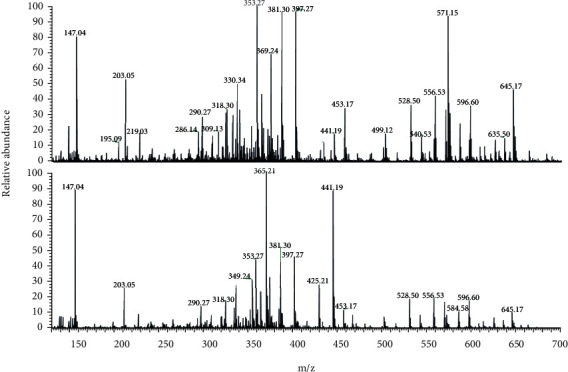
Mass spectrum (ESI(+)-MS)from cinnamon samples 15 (a) and 7 (b).

In Figure 3, it is possible to identify the chemical markers from the cinnamon samples, which are the compounds coumarin of *m/z* 147.04 [M+H] identified in high intensity and the cinnamaldehyde of *m/z* 133.06 [M+H], identified in low intensity [31]. The antioxidant compounds belonging to phenolic acids class (*m/z* 353.27) were detected in high concentration in the samples. Furthermore, it is possible to observe differences in ion intensity between samples 7 and 15 analyzed at the same concentration, which implies the variation of their metabolic content and antioxidant potential.

### 3.5. Study of Vascular Reactivity

Many studies indicate that antioxidant-rich food intake (mainly red wines, teas, seasoning, fruits, and vegetables) is linked with a protective action on the cardiovascular system in humans and animals [61, 62]. They produce, among several effects, vascular dilation in animals or patients, which could help control blood pressure levels [63, 64]. The present data showed that the both cinnamon samples tested induce a significant vasodilatory effect on isolated arteries at a very low concentration.

**Figure 4:**
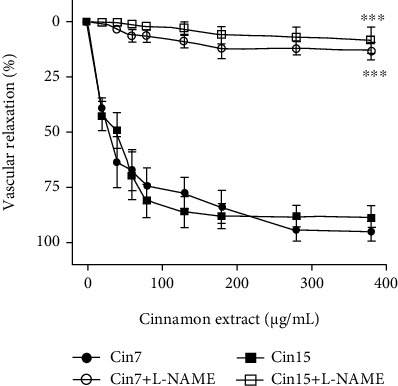
Vasodilatory effects of cinnamon samples 7 and 15 (cin7 and cin15) in endothelium-intact rat aortas precontracted with phenylephrine (0.1 *μ*M) in the absence or presence (30 min) of the nitric oxide synthase inhibitor L-NAME (100 *μ*M). The data points represent the mean ± SEM (*n* = 5 − 6) of the relaxing effect expressed as a percentage. Significant difference ^∗∗∗^*p* < 0.001.

As shown in Figure 4, the cinnamon samples 7 and 15 added cumulatively (0–380 *μ*g/mL) to the bath solution on the endothelium-intact arteries precontracted with phenylephrine-induced concentration-dependent vasodilation. The maximum effect for sample 7 (cin7) and sample 15 (cin15) was 95.8 ± 7.1% (*n* = 6) and 92.1 ± 6.4% (*n* = 6), respectively. This finding shows that endothelial nitric oxide production is the most important mediator to induce vascular dilation, since L-NAME (nitric oxide synthase inhibitor) almost abolished the vasorelaxant effect of both samples (8.2 ± 4.6%; *n* = 5 and 5.6 ± 3.8%; *n* = 5, respectively).

The reactive chlorine species are well known by their high injuriousness in the cardiovascular system, leading to endothelial cell dysfunction, chronic and local inflammation, and impairment of endothelium-dependent vasorelaxation [6, 65]. In various pathological disorders of the cardiovascular system that generate an active inflammation (e.g., diabetes, hypertension, infarction, and atherosclerosis), the reactive chlorine species (OCl^−^) can range high concentrations, reaching micromolar levels in the tissues affected and local circulation [30].

**Figure 5:**
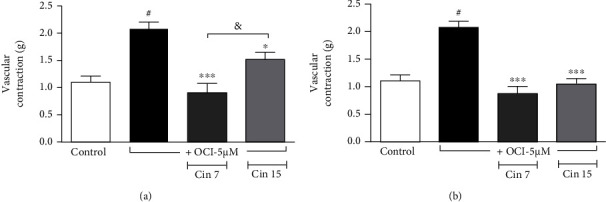
Vascular contraction induced by phenylephrine (0.1 *μ*M) in endothelium-intact rat aortas exposed or not to hypochlorite (5 *μ*M OCl^−^, 60 min). Some arteries were treated with cinnamon samples (cin7 and cin15) concomitantly to oxidative stimulus with OCl^−^ at different concentrations (a) 100 *μ*g/mL and (b) 500 *μ*g/mL. Vertical bars represent the mean ± SEM values of the maximum contractile effect (*n* = 5 − 7 for all protocols). Significant difference ^#^*p* < 0.001 compared to control. ^∗∗∗^*p* < 0.001 and ^∗^*p* < 0.05 compared to OCl^−^ treatment. ^&^*p* < 0.05 between groups cin7 and cin15.

In terms of vascular contraction, the treatment with OCl^−^ increased the contractile effect induced by adrenergic stimulation from 1.13 ± 0.12 g (*n* = 7) to 2.07 ± 0.13 g (*n* = 5). The concomitant treatment with cinnamon samples 7 and 15 at low concentration (100 *μ*g/mL) reduced the vascular contraction induced by phenylephrine to 0.90 ± 0.12 g (*n* = 6) and 1.51 ± 0.13 g (*n* = 6), respectively. However, the effect of sample 7 was more significant than sample 15, and it presented the level of contraction significantly smaller as compared to sample 15 (Figure 5(a)). At the highest concentration (500 *μ*g/mL) the two cinnamon samples inhibited the action of OCl^−^ with similar efficacy (Figure 5(b)).

## 4. Conclusions

This article evaluates the antioxidant capacity and activity of cinnamon, an important product worldwide consumed. This topic is very relevant, since the damage caused by free radicals is increasingly present in our population and can be reduced in our body by the ingestion of natural products rich in antioxidant compounds.

Cinnamon, although much studied for its antimicrobial property, is still little studied as a vasorelaxant. In addition, the electrochemical assays that generate the electrochemical index, as well as the use of a laccase-based biosensor from the fungus *Marasmiellus colocasiae* are unprecedented and therefore represent the innovation of the present work.

The antioxidant capacity of cinnamon samples was investigated through spectrophotometric and voltammetric methodologies. A high correlation coefficient of approximately 0.7 was found between EI and DPPH, ABTS, and Folin-Ciocalteu, which demonstrates a close relationship of the redox process of the antioxidant capacity in all methodologies. Electrochemical experiments evidenced high antioxidant quality of cinnamon due to the low oxidative potentials. Moreover, these results were significantly improved in a biosensor with the carbon paste-modified electrode using the laccase enzyme. Thus, these methodologies are efficient to study antioxidant capacity of natural products and emphasized the importance of cinnamon in a diet. The sample with the most potent antioxidant activity (7) and the least potent one (15) had similar vasodilatory activities, and both induce the nitric oxide production by endothelial cells. Furthermore, both samples tested reduced the oxidative injury evoked by OCl-, but sample 7 seems to be more efficient at low concentration.

## Figures and Tables

**Table 1:** Solvents for cinnamon extraction.

W/E	100 : 0 (*v*/*v*)	70 : 30 (*v*/*v*)	50 : 50 (*v*/*v*)	30 : 70 (*v*/*v*)	0 : 100 (*v*/*v*)
EI (*μ*A/V)^∗^	38.62 ± 28.4	50.17 ± 5.9	45.64 ± 46.5	57.33 ± 4.6	59.24 ± 24.2

W: percentage of water/E: percentage of ethanol. ^∗^Median values ± RSD.

**Table 2:** DPPH, ABTS, Folin AG, and EI correlation matrix for cinnamon species.

	ABTS	DPPH	Folin AG	EI
ABTS	1	0.83	0.5	0.62
DPPH	0.83	1	0.62	0.7
Folin AG	0.5	0.62	1	0.73
EI	0.62	0.7	0.73	1

**Table 3:** Percentage of decay for ABTS, DPPH, Folin, EI, and gallic acid equivalents for cinnamon species.

Sample	ABTS (% of decay)	SD	DPPH (% of decay)	SD	Folin (% of increase)	SD	EI (*μ*A/V)	SD	AG (g/g extract)	SD
1	96.73	4.84	67.20	3.36	0.24	0.07	7.77	0.39	0.09	0.04
2	69.86	3.49	62.61	3.13	0.35	0.05	6.13	0.31	0.07	0.00
3	80.64	4.03	65.42	3.27	0.70	0.09	11.86	0.59	0.17	0.07
4	94.06	4.70	74.30	3.71	0.51	0.05	6.07	0.30	0.06	0.02
5	88.75	4.44	70.55	3.53	0.32	0.08	7.37	0.37	0.08	0.07
6	90.52	4.53	73.85	3.69	0.77	0.12	7.37	0.37	0.08	0.00
7	97.55	4.88	79.03	3.95	0.87	0.03	12.84	0.64	0.19	0.05
8	95.05	4.75	83.29	4.16	0.94	0.06	8.81	0.44	0.11	0.00
9	97.02	4.85	77.86	3.89	0.55	0.04	9.14	0.46	0.12	0.04
10	75.92	3.80	60.91	3.05	0.59	0.04	6.20	0.31	0.06	0.05
11	96.42	4.82	71.63	3.58	0.89	0.06	9.47	0.47	0.12	0.06
12	47.33	2.37	41.78	2.09	0.37	0.02	4.73	0.24	0.03	0.00
13	92.71	4.64	81.89	4.09	0.48	0.07	8.38	0.42	0.10	0.01
14	68.67	3.43	51.11	2.56	0.06	0.00	3.39	0.17	0.02	0.00
15	72.39	3.62	41.69	2.08	0.34	0.06	4.93	0.25	0.03	0.02
16	82.32	4.12	51.58	2.58	0.59	0.00	4.98	0.25	0.03	0.02
17	87.13	4.36	59.10	2.95	0.65	0.06	6.68	0.33	0.07	0.02

**Table 4:** Antioxidant capacity of cinnamon samples using carbon paste modified electrode.

Sample	Unmodified electrodes (*μ*A)	SD	Modified electrodes (*μ*A)	SD	Signal increase (modified signal/unmodified signal)	Signal increase (%)
1	-0.47	0.34	-1.02	0.34	2.17	216.56
2	-0.67	0.56	-1.85	0.14	2.78	277.78
3	-1.01	0.72	-2.07	0.14	2.05	204.95
4	-0.93	0.62	-1.57	0.31	1.69	169.00
5	-0.68	1.08	-1.67	0.36	2.47	247.04
6	-1.23	0.01	-1.43	0.01	1.16	116.26
7	-0.72	0.848	-1.39	0.21	1.93	192.79
8	-0.57	0.52	-0.91	0.13	1.61	160.85
9	-0.47	0.41	-0.91	0.21	1.91	190.93
10	-0.50	0.66	-1.08	0.17	2.16	216.43
11	-0.75	0.19	-1.08	0.19	1.44	144.19
12	-0.68	0.60	-1.51	0.12	2.21	221.08
13	-0.66	0.55	-1.29	0.28	1.97	196.95
14	-0.70	0.45	-1.33	0.23	1.91	191.37
15	-0.61	0.26	-1.19	0.09	1.95	194.76
16	-0.38	2.19	-1.55	0.44	4.12	412.23
17	-1.13	4.96	-2.42	1.24	2.14	214.16

ANOVA test probability *p* = 6.12∗10^−6^ and *F* value = 38.41 shows that modified and unmodified values were statistically different.

**Table 5:** Metabolites present in cinnamon samples (*Cinnamomum*) with antioxidant property.

Ion mode	*m/z*	Empirical formula	Compound name	Chemical structure	Reference
ESI(+)MS	147.04	C_9_H_6_O_2_	Coumarin	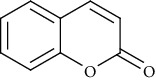	[44, 45]
ESI(+)MS	133.06	C_9_H_8_O	Cinnamaldehyde	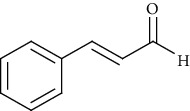	[46, 47]
ESI(+)MS	181.07	C_9_H_8_O_4_	Caffeic acid	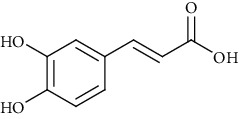	[48]
ESI(+)MS	169.05	C_8_H_8_O_4_	Vanillic acid	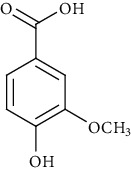	[49]
ESI(+)MS	453.17	C_21_H_24_O_11_	Catechin 3′-glucoside	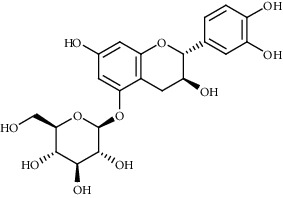	[50]
ESI(+)MS	645.16	C_27_H_32_O_18_	Myricetin derivative	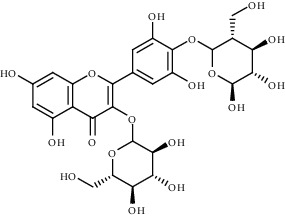	[51]
ESI(+)MS	317.21	C_16_H_12_O_7_	Isorhamnetin	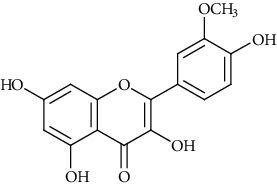	[50, 52]
ESI(+)MS	369.24	C_21_H_20_O_6_	Curcumin	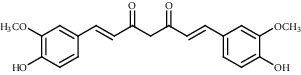	[53]
ESI(-)MS	165.06	C_10_H_12_O_2_	Eugenol	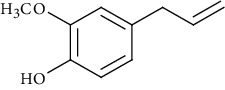	[44, 54, 55]
ESI(-)MS	195.05	C_10_H_10_O_4_	Ferulic acid	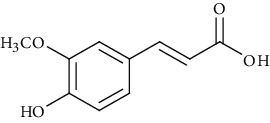	[56, 57]
ESI(+)MS	153.02	C_7_H_6_O_4_	Protocatechuic acid	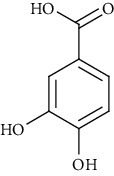	[56]
ESI(-)MS	191.06	C_11_H_12_O_3_	Coumaryl acetate	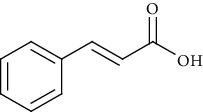	[58]
ESI(-)MS	147.04	C_9_H_8_O_2_	Cinnamic acid	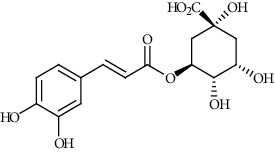	[59]
ESI(-)MS	353.09	C_16_H_18_O_9_	Chlorogenic acid	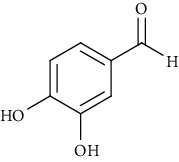	[60]
ESI(-)MS	137.02	C_7_H_6_O_3_	3,4-Dihydroxybenzaldehyde	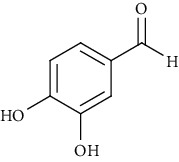	[60]

## Data Availability

The authors confirm that the data supporting the findings of this study are available within the article, whereas, any doubt can be requested from the corresponding author.
